# The economic analysis of two treatment procedures for incisional hernias - alloplastic versus tissular

**Published:** 2014-03-25

**Authors:** C Mavrodin, G Pariza, D Ion, M Ciurea

**Affiliations:** Emergency Hospital Bucharest

**Keywords:** incisional hernia, alloplastic surgery, tissular surgery

## Abstract

Incisional hernias are a common complication of abdominal surgery. Research shows that their incidence reaches 10%-11% of the total number of patients subject to laparotomy. Recurrent hernias are the main complication of eventrations and its rate ranges from 5 to 54%, depending on both the surgical procedure used and the follow-up methods. The goal of this study is the comparative cost analysis of two procedures used in the treatment of event rations, tissular versus alloplastic, the former, leading very often to recurrence requiring a new surgical intervention. The analysis comprised 156 cases of surgeries performed for incisional hernia in 2007 in the clinic of Surgery III, SUUB (Bucharest University Emergency Hospital). Tissular procedures were used in 42 cases and prosthetic procedures in 114 cases. The medium-term postoperative follow-up has revealed 17 relapses (40.4%) in the tissular batch and no relapse in the batch where parietal prosthesis was used. If the short-term costs of the tissular procedures are low as compared with the prosthetic procedures, on the medium-term the costs increase by 24.35% due to the high rate of relapses of tissular procedures. Therefore, the tissular procedure must be abandoned due to the high rate of relapse, as this drives additional costs required for the alloplastic repair of the abdominal parietal defects in a subsequent surgical intervention.

## Introduction

Incisional hernias are common conditions whose treatment is specific to the general surgery. Considered nonspecific conditions, their importance is often disregarded. Without exaggerating their degree of severity, they must be treated with proper attention, otherwise they result in severe complications for the patient. 

 Over time, various surgical procedures have been considered for the treatment of incisional hernias, according to the level of the existing medical knowledge and to the development of materials and technologies used. Currently, the use of alloplastic materials in the prosthetic repair of incisional hernias is without doubt a mandatory requirement for most cases [**1**]. 

 Incisional hernias are a common complication of abdominal surgery. Research shows that their incidence reaches 10% -11% of the total number of patients subject to laparotomy. Eventrations are an important morbidity source as they complicate by incarceration in 6-15% of cases and by strangulation in 2% of cases. The effects can be related with a mortality rate of 10% if the surgical intervention is not prompt [**2**].

 Although various procedures for the repair of these parietal defects have been considered, the results are often disappointing. Following the use of tissular procedures, the relapse rate ranges between 24% and 54% while the use of prosthetic materials in the surgery of parietal defects reports a relapse rate ranging between 1.5 and 10% [**2,3**]. We notice the progress during the past few years regarding the technical and tactical methods for solving incisional hernias as well as the diversity of prosthetic devices used within this pathology for the repair of the defects of the abdominal wall. They are beyond the imagination of doctors, as the doctor often faces a difficult decision by having to opt for one of the options. The disappointing outcomes of the tissular procedures require that they must be completely abandoned and the prosthetic approach of the eventrations must be considered mandatory.

 Although the therapy of incisional hernia through tissular procedures is still used in 10%-15% of the cases, most of the technical procedures currently used involve the use of a synthetic prosthetic biomaterial [**4**]. 

 Recurrent hernia is the main complication of eventrations and is obviously the consequence of the use of an unsatisfactory surgical procedure. It has been proven that the relapse rate increases with the number of previous interventions underwent by the patient. The scientific literature states that this ranges from 5 to 54%, depending on both the surgical procedure used and the follow-up methods [**3-7**]

 Its high incidence has required caution in the selection of the surgical treatment of incisional hernia. Alloplastic methods have replaced the tissular procedures as the latter are reserved to surgical emergencies, when the required prosthetic materials cannot be supplied due to financial reasons.

 The goal of this study is the comparative cost analysis of two procedures used in the treatment of eventrations, tissular versus alloplastic, the former leading very often to recurrence requiring a new surgical intervention.

## Method

This is a descriptive, cross-sectional study and consists of the analysis of direct costs related to the two procedures for the treatment of eventrations.

 The following phases have been covered in order to meet the purpose:

 a. identification of the main therapeutic phases for each type of intervention

 b. identification of resources - categories and quantities required for each procedure

 c. identification of costs per physical unit;

 d. identification of direct costs for every therapeutic phase in both alternatives

 e. identification of the overall cost.

 f. comparison of long-term costs - 2 years

 a. Therapeutic phases:

 The following distinct phases have been considered for each type of intervention: preoperative phase, operative phase, general anaesthesia, immediate postoperative phase and follow-up after 2 years.

 b. Identification of resources required in every phase, per categories of resources and per quantities required 

 The following categories of resources have been determined: paraclinical and imaging investigations, medicines, medical materials, accommodation (days of hospitalization) and salary costs directly related to the medical personnel involved in the surgical intervention (labour costs).

 c. Identification of costs per physical unit:

 Only the direct costs of the healthcare unit have been considered. 

 The following were not considered: indirect costs, incurred by the patient or the family (due to estimate difficulties), the costs of the sickness benefit (71% of the patients undergoing surgery from 2007 to 2009 were retired persons), human resources costs.

 The following sources were considered for the calculation of costs:

 - the purchase costs per unit used by the hospital at the last public procurement of the relevant product

 - rates covered by the National Health Insurance Fund

 - the cost per day of hospitalization, calculated by the hospital

 d. Identification of direct costs for every therapeutic phase in both alternatives

 The following formulas have been used:

 Cost by type of resources = quantity (physical units) x unit cost

 Cost by category of resources = ∑ costs by type of resources

 Cost by phase = ∑ costs by categories of resources in every phase

 e. Identification of the overall cost

 Cost by procedure = ∑ costs by phases for all the phases of a procedure

 f. Comparison of overall costs by alternatives

 The overall costs have been compared directly and as percentage differences.

 The percentage difference has been calculated according to the formula

 (Cost version A – Cost version B) x 100 / Cost version B

 The percentage difference shows how much difference is between the costs of the two procedures against the cost of the cheapest procedure.

## Results

Comparison of costs by therapeutic phases

 The preoperative phase has identical costs in both situations.

 The operative phase has been 27.72% cheaper when using the tissular approach vs. the prosthetic approach. The cost by components shows a high difference due to the medical materials, as the alloplastic procedure includes the cost of the Prolene prosthesis and of the stitches.

 The general anaesthesia phase has been 9.20% cheaper when using the tissular approach vs. the prosthetic approach, as the difference comes from the cost of medicines due to a longer duration of the intervention for the alloplastic procedure.

 The postoperative phase has been 9.19% cheaper when using the tissular approach vs. the prosthetic approach (**Table 1**).

**Table 1:** 

Intervention type/Costs	Tissular procedure	Alloplastic procedure
Cost of preoperative phase - RON -	750	750
Cost of operative phase - RON -	1701.86	2354.66
Cost of general anaesthesia phase - RON -	302.62	333.29
Cost of postoperative phase - RON -	2042.60	2249.35
Overall costs of intervention - RON -	4797.08	5687.30

**Fig. 1:**
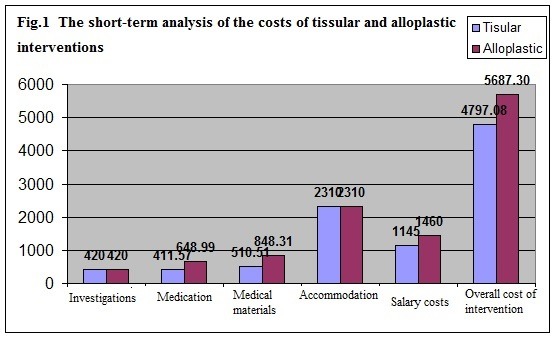
The short term analysis of the costs of tissular and alloplastic interventions

 The short-term analysis of the costs of tissular and alloplastic interventions

 The category of resources with the highest percent impact on the cost difference relates to medical materials within the operative phase and to medicines within the general anaesthesia and the postoperative phase.

 Overall, the estimate cost of alloplastic prosthetic repair amounts to RON 5687.30, 15.65% higher than the cost of the tissular approach, with an estimate cost of RON 4797.08. In terms of the cost, a short-term analysis of the use of the two procedures recommends the use of the tissular procedure (**Fig. 1**).

 The medium-term analysis of the costs of tissular and alloplastic interventions

 In 2007, the Department of Surgery III - SUUB performed surgeries on 156 incisional hernias, out of which 114 through alloplasty with polypropylene monofilament prostheses and 42 through tissular procedures. The medium-term analysis of the 156 interventions shows that, from 2008 to 2009, 17 relapses have been reported among the 42 cases treated through the tissular procedure, vs. no relapse among the 114 cases treated through alloplasty. Therefore, within the clinic of Surgery III SUUB, the relapses related to eventrations subject to surgery through tissular procedure amounted to 40.47% of the total number of these interventions.

 According to the scientific literature, relapses related to tissular procedures may even amount to 63%, depending both on the procedure used and on the methods of follow-up of patients [**7,9,12**].

 Thus, even if the initial assessment of costs shows that the tissular procedure should be preferred, relapse occurring in such a short time, in a significant percent, prohibits its use as surgical method both in terms of the patient’s benefit and in terms of costs.

 The analysis of tissular procedure costs after 2 years shows they are in fact 24.35% higher than prosthetic procedures due to the fact that another intervention is required in over 40% of cases and the alloplastic procedure is used. Table 8 shows the short- and medium-term costs for a sample of 100 patients on which both the tissular and the alloplastic procedures were used, considering the analysis of relapses within the clinic of Surgery III SUUB (**Table 2**).

**Table 2:** Overall costs by types of interventions - short and medium term

Intervention type	Tissular procedure	Alloplastic procedure
Short-term cost in 100 patients	479708.31	568730.21
Medium-term cost of relapses in 40 patients	227492.08	0.00
Overall medium-term costs	707200.39	568730.21
Short-term cost/patient	4797.08	5687.30
Medium-term cost/patient	7072.00	5687.30
Short-term % tissular vs. alloplastic		-15.65%
Medium-term % tissular vs alloplastic		-15.65%

**Fig. 2:**
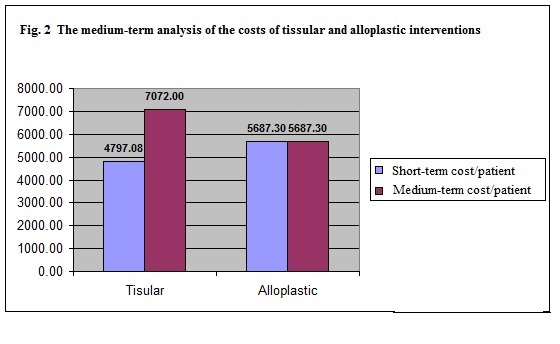
The medium term analysis of the costd of tissular and alloplastic interventions

 The highest percent difference of costs seems to occur within the medium-term assessment (2 years) (24.35% more expensive in the tissular approach) (**Fig. 2**).

## Discussions

 Despite the progress of prosthetic materials and of modern surgery, the selection of the surgical procedure of incisional hernias is and remains a controversial issue, as it depends on the surgeon preferences or on the financial resources of the hospital. The large number of this kind of interventions, as it is difficult to decrease incidence of incisional hernias, results in the increase of costs in the healthcare system. The selection of a procedure must also consider the long-term progress of patients, as a high relapse risk leads to the increase of the overall costs, even if it seems cheaper in the first phase (e.g. tissular procedures).

 According to the data supplied by the National Institute of Public Health, in 2007 the frequency of surgical interventions for incisional hernias amounted to 9399 interventions/year, out of which 5512 tissular procedures (code 30403-00/J12901) and 3887 prosthetic procedures (code 30405-01/J12903), summing up over 80 000 days of hospitalization/year. It is noticed the prevalence of tissular procedures at national level, as they increase significantly the costs of the healthcare system.

 According to the literature and to the experience of the clinic, the alloplastic parietal prosthetic repair has the following reliable benefits for the patient: 

 - Current prosthetic materials of highest quality and clinically effective are currently affordable even under the financial conditions of our patients; thus, the financial reason is not justified as it hides the lack of interest in selecting a modern and effective surgical method [**1,5,13-16**].

- A much lower rate of postoperative complications at distance - the relapse of eventration, enables the patients who underwent alloplastic surgery to remain integrated in the initial social and professional activity.

- The analysis of costs has shown that the tissular approach is 15.65% cheaper than the prosthetic approach (RON 5687.30 vs. RON 4797.08).

- The operative phase has been 27.72% cheaper when using the tissular approach vs. the prosthetic approach.

-The cost difference in the operative phase is entirely determined by the medical materials used and by the direct salary costs related to the medical staff involved in the surgical intervention (labour costs).

- The highest cost differences occur in the medium-term assessment showing that the tissular approach requires 24.35% more money that the prosthetic approach.

- In the medium-term assessment, the cost difference is determined by the need of a new intervention and by the mandatory use of prosthetic procedures for all relapses (40% of the studied batch).

- Under these circumstances, corroborated with the data from literature regarding the lower occurrence of complications in patients who underwent alloplastic surgery, it is recommended for both the hospital policy - as health care provider, and the policy of the National Health Fund, as service payer, to encourage the predominant use of prosthetic procedure in the treatment of incisional hernias. 

## Conclusions

 The conclusions show the need for surgical judgement in the correct selection of the surgical procedure. The tissular procedure must be completely abandoned due to the high rate of relapse, as this drives additional costs required for the alloplastic repair of the abdominal parietal defects in a subsequent surgical intervention.
